# Breakthrough Cancer Pain: Review of Prevalence, Characteristics and Management

**DOI:** 10.4103/0973-1075.53506

**Published:** 2009

**Authors:** Seema Mishra, Sushma Bhatnagar, Prakash Chaudhary, Shiv Pratap Singh Rana

**Affiliations:** Dr. B.R.A, IRCH, AIIMS, Ansari Nagar, New Delhi, India

**Keywords:** Analgesic ladder, Breakthrough pain, Cancer, Cancer pain

## Abstract

Breakthrough pain has been associated with a reduced likelihood of adequate pain control. Despite the large and variable incidence of this phenomenon due to varied definitions of this type of pain, only a few studies have been conducted to assess and effectively treat breakthrough pain though the importance of managing breakthrough pain has been acknowledged by all. A large number of drugs from various classes and novel methods of administration like nasal and transmucosal buccal route, as in the case of fentanyl, have been used in these studies to manage this type of pain. A drug is needed with a quick onset of action and optimal duration that matches the characteristics of breakthrough pain. Some steps have been taken in earlier studies which used nasal formulation of fentanyl as it was found to achieve adequate and quick pain relief. However, further studies are required to confirm this so that in the future we can have as effective protocols for managing breakthrough pain as we have today for managing persistent pain as given by the World Health Organization.

## INTRODUCTION

Pain is one of the most common and feared symptoms of cancer. Many patients suffering from cancer have more than one type of pain, not all pains are, however, due to cancer itself. At least two-third of the patients suffering from advanced cancer report pain (WHO 1996). Pain relief is achieved adequately in a majority of cancer patients using the WHO guidelines.[1–3] Pain in cancer patients has two components. One is persistent pain that lasts for more than 12 hours/day. However, in addition to persistent pain, patients may also experience transient exacerbations of significant and severe pain on a background of otherwise well controlled pain. These severe flare ups of pain are called breakthrough pain as the pain breaks through the regular pain medication. Specific characteristics that further define breakthrough pain include it's relation to the fixed dose of opioid medication, temporal features, precipitating events and its predictability. This breakthrough pain has an incidence of about 40-86% as reported in various studies.[3–10] The differences in incidence, as reported in various studies, are probably due to different settings and meaning attributed to the term breakthrough pain. However, with proper evaluation and treatment, breakthrough pain can be managed successfully just like persistent pain.[9] The aim of this article is to review the past studies and literature written and conducted on breakthrough pain.

## TERMINOLOGY AND TYPES OF BREAKTHROUGH PAIN

An international survey of cancer pain characteristics indicated that the breakthrough pain definition varied from country to country.[10] Breakthrough pain is an English term with no literal translation in many languages, including Spanish, Italian, and Portuguese, among others.[7,8] There is not a single definition of breakthrough pain to which all researchers agree with. For example, in United Kingdom, this term is used as a sign of end of dose failure during dose titration for pain management.[3] To avoid confusion, some experts have advocated the use of broader terms like episodic pain or transient pain in place of breakthrough pain, whereas some have listed the types of breakthrough pain depending on its predictability and precipitating factors.[10]

Following are the types of breakthrough pain:

- Idiopathic
- Incidental
- End of Dose

### Idiopathic pain (Spontaneous)

Stimulus independent i.e no obvious precipitating factor. Pain comes on without warning and has no precipitating stimulus. Sudden, sharp, and often marked by a disabling crescendo, idiopathic pain is common in neuropathic pain condition.[11]

### Incidental pain

*Incident pain* has an identifiable cause. The cause can be *volitional*, as in pain caused when the patient initiates movement such as walking, or *nonvolitional*, as in the type of pain that can occur during bladder spasm after voiding. The most common type of BTP in cancer patients is incident pain related to bone metastases, but cancer patients are also subject to sudden paroxysmal pain associated with neuropathic origins.

### End of dose pain

It results when the dose of drug drops below the analgesic level. End of dose pain occurs with greater frequency at the end of dosing interval of around the clock opioid medication.[12]

## CHARACTERISTICS OF BREAKTHROUGH PAIN

It is critical that breakthrough pain be differentiated from persistent pain. Although, conventionally, persistent pain is defined as pain lasting for 12 or greater than 12 hours/day, there are certain other factors that characterize breakthrough pain like - it's relation to fixed opioid doses being used for persistent pain, temporal features, precipitating events and predictability.

End of dose pain occurs with greater frequency at the end of dosing interval of around the clock opioid medication. This can be linked to the dissipating effect of analgesia near the next scheduled opioid dose. This type of pain has an incidence of about 13.2%-33% as reported in various studies.[5,6,10]

Temporal features include the onset, duration and frequency of each breakthrough pain episode. The onset is sudden reaching a maximum intensity within one second to 30 minutes with a median of about three minutes.[3–6,10] The number of episodes per day varies from 1-3600/day as reported in different studies with an average of four to six episodes/day.[5,6] The duration of each episode lasted from 1-240 minutes with a median duration of 15-30 minutes.[5,6,10]

Episodes of breakthrough pain may or may not be associated with a precipitating factor and therefore may or may not be predictable. Precipitating factors can be identified in 55%-80% episodes whereas no cause is apparent in 27-38.3% of breakthrough pains.[3,4,6] 48.2%-57% episodes are never predictable, whereas only 12-15.3% episodes of breakthrough pain were always predictable.[5,6,10]

The cause of breakthrough pain also differs from patient to patient. Studies show that 67-76% pains are caused by the neoplasm itself, 20-33% episodes are due to the treatment received, wheras up to four per cent breakthrough pains are of uncertain cause.[5,6]

## PAIN CATEGORIZATION

The underlying mechanism of breakthrough pain may be nociceptive, neuropathic or mixed.[10,13] Nociceptive pain may be somatic due to involvement of somatic structures like bone or muscle; or visceral if due to involvement of underlying solid or hollow viscus. Somatic pain is aching or throbbing in type whereas visceral pain is described as squeezing, gnawing or crampy in type. Neuropathic pains are due to involvement of peripheral or central afferent neural pathways and described as burning or lancinating in character. The incidence of nociceptive pain is 38-53%, neuropathic pain is 10-54% whereas mixed pain occurs in about 20-52% patients.[5,6,10]

## ASSESSMENT OF BREAKTHROUGH PAIN

Assessment of pain is done using pain questionaires and patient related instruments.[5,8,10,13]

*Pain Questionaires* are usually administered as a structured interview and includes questions about breakthrough pain intensity, number of episodes/day, types of breakthrough pain, temporal features, precipitating factors and predictability after assessing the answers, the etiology and path physiology of breakthrough pain is identified.

### Patient related instruments

Memorial pain assessment card measures pain and global mood using three 100mm visual analogue scales (VAS)-pain intensity, pain relief and mood respectively and an eight item verbal rating scale. The score on mood VAS tells about the psychological distress.

Brief Pain Inventory (BPI)–It is a widely used tool to assess pain intensity with a numerical 0-10 scale where 0 means no pain and 10 would mean worst pain possible. A seven item subscale of BPI evaluates the degree to which pain interferes with function and quality of life. Each activity is assessed on a 0-10 numeric scale. (0-no interference, 10-complete interference)

Back Depression Inventory is a 21 item self report instrument designed to measure the symptoms associated with depression. Beck Anxiety Inventory is a similar instrument designed to measures the symptoms associated with anxiety.

Karnofsky Performance Status score is an observer rated measure of performance status. The score indicates the ability to function physically.

FACT-G is a 28-item general patient rated measure of quality of life. Each item is scored from 0 to 4 and anchored from ‘not at all’ to ‘very much’

MSAS-SF is a validated patient rated instrument that includes patient assessment for symptom frequency or distress for 32 highly prevalent physical and psychological symptoms. All the above mentioned instruments have been used in studies conducted in the past for qualitative assessment of breakthrough pain.

## CONSEQUENCES OF BREAKTHROUGH PAIN

Untreated breakthrough pain has significant consequences for individual patients, their caregivers and the healthcare system. Without treatment, flares of breakthrough pain can harm a person's sense of well being, interfere with daily activities, interrupt disease related treatment schedules and make it even more difficult to treat persistent pain. As fear of breakthrough pain events grows, patients tend to remain sedentary thus exacerbating physical deconditioning and pain related disability.[14]

Effective treatment of breakthrough pain is not only good practice but also cost effective as past studies have shown that effective breakthrough pain relief decreases cost of overall treatment by five times. Therefore, although assessment of breakthrough pain and its treatment may initially increase the cost of treatment, overall it will be less costly.

## MANAGEMENT

A large number of studies conducted in the past have acknowledged the need for treating breakthrough pain, but not many have gone beyond the use of supplemental doses of opioid. It is only in the past few years that use of some other drug and novel route of administration has been studied to relieve breakthrough pain.

Four principles can be followed to guide management of breakthrough pain.

1. Importance should be given to careful assessment of breakthrough pain to evaluate its characteristics. It should also determine the etiology, pathophysiology and pain-patient's overall clinical status relation.
2. Consideration should be given to the primary treatment of underlying etiology. This may involve surgery to decompress obstructed bowels, surgery to repair fracture or radiotherapy for painful metastases.
3. Adjustments in the dose of regular (usually opioid) analgesic must be considered given the relation between occurrence of breakthrough pain and baseline analgesic regimen.
4. Primary analgesic approach to treatment of breakthrough pain must be reevaluated if required. For instance most popular approach is the use of supplemental dose of opioid as and when required, but usually neither the pharmacokinetics nor the pharmacodynamics profile of the drug used matches the desired requirements. The ideal choice would be one with a fast onset of action and a short half life. A large number of drugs and approaches have been used to treat breakthrough pain

## PHARMACOLOGICAL METHODS

### Anti-inflammatory drugs

Additive analgesia produced by NSAIDS and steroidal anti-inflammatory drugs is useful in painful bone metastases, mucosal and skin lesions. While long acting NSAIDS allowing once or twice daily dosing is preferred in patients taking multiple drugs, rescue doses of particular formulations of NSAIDS (sublingually or parentally)is preferred in treating breakthrough pain particularly when side effects from rescue doses of opioids become intolerable.

### Opioids

Patients with breakthrough pain are usually treated with an opioid drug. The use of as needed analgesia with rescue doses of opioids to treat established breakthrough pain or prevent anticipated episode is the current gold standard of management in spite of the fact that the pharmacokinetics of oral opioid does not match the requirements of breakthrough pain.[15–17] A large number of routes are available for opioid administration.

### Oral

Typically, the rescue doses consist of an immediate release preparation that is the same dose as being administered on around the clock basis although the most effective dose remains unknown. Titration of the rescue dose according to the character of breakthrough pain is, therefore, advocated to identify the suitable dose.

### Sublingual

This route has limited application due to lack of formulations, poor absorption of drugs and inability to deliver high doses that are prevented by swallowing.

### Rectal

Rectal administration offers the possible pharmacokinetic advantage of bypassing first pass metabolism by direct ally entering the systemic circulation via the lower rectal veins. But there is no clear demarcation between portal and systemic drainage and this may render proportion of drug absorbed through portal system difficult to predict. Therefore, a considerable difference in bio-availability of rectally administered morphine has been observed in between individuals.

### Transmucosal

Both oral transmucosal and nasal formulations of fentanyl have become available and studied recently for relief of breakthrough pain. The efficacy of oral fentanyl was compared with morphine sulfate immediate release oral form and it was found that pain relief was earlier and quantitatively better with former.[12,13] The dose of oral fentanyl used varied from 200-1600 μg. Nasal fentanyl spray 20ug was also found to be better than oral morphine to relieve breakthrough pain.[17]

### Subcutaneous and intravenous route

Parenteral route is best for immediate pain relief. Subcutaneous route is equally efficacious although onset is slower than intravenous route.

Previous studies have mainly studied the benefits of morphine sulfate immediate release (MSIR) and fentanyl citrate for the management of breakthrough pain. In one study, oral transmucosal fentanyl citrate (OTFC) was used and pain relief (PR) was measured at 15, 30, 60 minutes post intake. The dose of OTFC varied from 200-1600 μg, the exact dose being decided during the drug titration phase. By exploratory analysis, it was concluded that OTFC provided earlier and better PR than MSIR that was being used by the patients before they entered into this study.[15]

However, one study directly compared the effect of MSIR versus OTFC and concluded that pain intensity, pain relief and global performance of medication scores were significantly better for OTFC.[16]

Nasal fentanyl was used in one study (20 ug)to treat breakthrough pain. It was concluded that 75% patients had better or same pain relief as compared to MSIR that they were using earlier. 33% patients had pain relief within 5 minutes and 75% patients said that they would continue to take nasal fentanyl in preference to MSIR.[17]

### Adjuvant

The regular uses of antidepressants, antiarrhythmics and anticonvulsants have been used to treat pain refractory to opioids and particularly neuropathic pain.

### Miscellaneous

Spasmolytics like octreotide are used to treat colicky pain and drugs like bisphosphonates are used to treat metastatic bone disease.

### Non-pharmacological methods

Physiatric techniques like physical therapy or use of orthotics are useful in musculoskeletal pain; bracing is of value in movement related pain. Psychological techniques are useful in certain patients.

### Invasive measures

Anaesthetic approaches useful in treatment of persistent pain are sometimes useful to treat breakthrough pain like chemical neurolysis and epidural catheter infusion of local anaesthetics, opioids, and clonidine.[18–20]

A percutaneous cordotomy is useful as a last resort to treat refractory incident pain from bone metastasis. Intrathecal phenol block and pituitary ablation have also been used to treat refractory breakthrough pain.

The results of these invasive procedures are often sub-optimal when considering the risk of side effects.[21]

## CONCLUSION

Breakthrough pain has been associated with a reduced likelihood of adequate pain control. Despite the large and variable incidence of this phenomenon due to varied definitions of this type of pain, only a few studies have been conducted to assess and effectively treat breakthrough pain. However the importance of managing breakthrough pain is acknowledged by all. A large number of drugs from various classes and novel methods of administration like nasal and transmucosal buccal route as in case of fentanyl have been used in these studies to manage this type of pain. A drug is needed with a quick onset of action and optimal duration that matches the characteristics of breakthrough pain. Some steps have been taken in earlier studies which used nasal formulation of fentanyl as it was found to achieve adequate and quick pain relief. However, further studies are required to confirm this so that we can have as effective protocols to manage breakthrough pain as we have today for managing persistent pain, as given by WHO.
